# Assessing Phospholipase A_2_ Activity toward Cardiolipin by Mass Spectrometry

**DOI:** 10.1371/journal.pone.0059267

**Published:** 2013-03-22

**Authors:** Yuan-Hao Hsu, Darren S. Dumlao, Jian Cao, Edward A. Dennis

**Affiliations:** 1 Department of Chemistry, Tunghai University, Taichung, Taiwan; 2 Department of Pharmacology, University of California San Diego, La Jolla, California, United States of America; 3 Department of Chemistry and Biochemistry, University of California San Diego, La Jolla, California, United States of America; Karolinska Institutet, Sweden

## Abstract

Cardiolipin, a major component of mitochondria, is critical for mitochondrial functioning including the regulation of cytochrome *c* release during apoptosis and proper electron transport. Mitochondrial cardiolipin with its unique bulky amphipathic structure is a potential substrate for phospholipase A_2_ (PLA_2_) *in vivo*. We have developed mass spectrometric methodology for analyzing PLA_2_ activity toward various cardiolipin forms and demonstrate that cardiolipin is a substrate for sPLA_2_, cPLA_2_ and iPLA_2_, but not for Lp-PLA_2_. Our results also show that none of these PLA_2_s have significant PLA_1_ activities toward dilyso-cardiolipin. To understand the mechanism of cardiolipin hydrolysis by PLA_2_, we also quantified the release of monolyso-cardiolipin and dilyso-cardiolipin in the PLA_2_ assays. The sPLA_2_s caused an accumulation of dilyso-cardiolipin, in contrast to iPLA_2_ which caused an accumulation of monolyso-cardiolipin. Moreover, cardiolipin inhibits iPLA_2_ and cPLA_2_, and activates sPLA_2_ at low mol fractions in mixed micelles of Triton X-100 with the substrate 1-palmitoyl-2-arachidonyl-*sn*-phosphtidylcholine. Thus, cardiolipin functions as both a substrate and a regulator of PLA_2_ activity and the ability to assay the various forms of PLA_2_ is important in understanding its function.

## Introduction

Over the past 40 years, scientists have devoted themselves to understanding the enzymes that can hydrolyze the *sn*-2 ester bond of phospholipids to release free fatty acids [1], leading to the discovery of the phospholipase A_2_ (PLA_2_) superfamily. The free fatty acids released by PLA_2_ can be converted to eicosanoids, including the prostaglandins and leukotrienes, which are involved in inflammatory responses [2]. So far, six types including sixteen distinct groups of PLA_2_ have been identified (Table 1). The three main types of PLA_2_, secreted PLA_2_ (sPLA_2_), cytosolic PLA_2_ (cPLA_2_) and Ca^2+^-independent PLA_2_ (iPLA_2_), were found early and are well-studied; hence several enzymes have been categorized for each type. Platelet activating factor acetylhydrolase (PAF-AH), lysosomal PLA_2_ and adipose-specific PLA_2_ (Ad-PLA_2_) were later discovered and are now part of the phospholipase A_2_ superfamily. These PLA_2_s can be differentiated by their cellular localization, Ca^2+^ dependence of the activity and their physical properties [3]. The activities and membrane association of sPLA_2_(s) and cPLA_2_(s) are Ca^2+^ dependent, whereas the activity of iPLA_2_, Lp-PLA_2_, lysosomal PLA_2_ and Ad-PLA_2_ are independent of Ca^2+^ [1]. The activation of these PLA_2_s have been vigorously investigated and shown to be involved in inflammation, atherosclerosis, cancer, diabetes and neurodegenerative diseases.

**Table 1:** The phospholipase A_2_ superfamily.

Type	Group	Subgroups	Size (kDa)	Ca^2+^	Cellular Location	Catalytic Residues
	GI	A, B	13–15			
	GII	A, B, C, D, E, F	13–17			
	GIII		15–18			
	GV		14			
sPLA_2_	GIX		14	Yes	Secreted	His/Asp
	GX		14			
	GXI		12–13			
	GXII		19			
	GXIII		<10			
	GXIV		13–19			
cPLA_2_	GIV	A(α), B(β), C(γ)	60–114	Yes	Cytosol	Ser/Asp
iPLA_2_	GVI	A, B, C, D, E, F	84–90	No	Cytosol Mito.	Ser/Asp
PAF-AH	GVII	A(Lp-PLA_2_), B(PAF-AH II)	40–45	No	LDL, HDL	Ser/His/Asp
	GVIII	α_1_, α_2_, β	26–40			
Lysosomal PLA_2_	GXV		45	No	Lysosome	Ser/His/Asp
Adipose-specific PLA_2_ (AdPLA_2_)	GXVI		18	No	Adipocyte	His/Cys

Adapted from Dennis et al [1].

The complex anionic phospholipid, cardiolipin, is a major component of the inner membrane of mitochondria and bacterial membranes [4], [5], [6]. Cardiolipin has been shown to be a substrate of both sPLA_2_ and cPLA_2_ utilizing a fluorescent labeled cardiolipin [7], [8] and implied to be a substrate for iPLA_2_ [9]. Most cardiolipin resides in mitochondria and accounts for 10–20% of the total lipid in mammalian cells. Cardiolipin is an important membrane component maintaining the integrity of mitochondria and is critical for the production of ATP via the electron transport chain [10], [11], [12] by stabilizing the electron transport chain complex in the inner mitochondrial membrane [13], [14]. Cardiolipin associates with the membrane-anchored cytochrome *c* under homeostatic conditions [15]. Oxidative stress causes peroxidation of cardiolipin and thus, hampers the electron transport chain and alters mitochondrial bioenergetics [16], [17], [18]. When programmed cell death is initiated, the interactions between cytochrome *c* and cardiolipin is disturbed [15], [19], [20]. Besides its functions in mitochondria, cardiolipin has been shown to be associated with several diseases. Cardiolipin causes the immune response to the anti-cardiolipin antibodies, which has been shown to be associated with increased risks of venous or arterial thrombosis and ischemic coronary and cerebral disease [21], [22], [23], [24]. Cardiolipin is also a significant and normal physiologic component present in human plasma lipoproteins including LDL and HDL [25]. Recently, cardiolipin release from damaged mitochondria has been shown to exacerbate breathing problems of pneumonia patients [26]. Uniquely and more importantly, cardiolipin is a substrate for the PLA_2_ superfamily of enzymes; however, their activities *in vitro* have not yet been characterized.

An X-linked tafazzin gene mutation causes the alterations of mitochondrial cardiolipin, including modified acyl chains and accumulation of monolyso-cardiolipin, and with a deficiency in cardiolipin remodeling, many patients further develop Barth syndrome. [9], [27], [28]. The 85-kDa GVIA iPLA_2_ (also known as iPLA_2_β) has been shown to localize to mitochondria in many types of cells [29], [30], [31], [32], which indicates iPLA_2_ is localized in the proximity of cardiolipin. iPLA_2_ was characterized and it showed potent phospholipase, lysophospholipase and transacylase activities toward phosphatidylcholine (PC) which could be blocked by various inhibitors [33], [34], [35], [36]. iPLA_2_ has been suggested to be responsible for cardiolipin deacylation and monolyso-cardiolipin accumulation in Barth syndrome and hypertensive heart failure [9], [37]. Inhibition of iPLA_2_ can suppress the phenotype of tafazzin knockouts in drosophila [9]. The discovery that cardiolipin is involved in Barth syndrome has suggested that the catabolism of cardiolipin by PLA_2_ plays a pivotal role in mitochondria maintenance. iPLA_2_ inhibition could be a potential treatment for Barth syndrome patients.

Because radiolabeled cardiolipin is challenging to synthesize, our goal in this study was to develop a mass spectrometry methodology to quantitate PLA_2_ activity toward natural and synthetic non-radiolabeled cardiolipin and further understand the activity of PLA_2_ toward cardiolipin. We utilized mass spectrometry (GC/MS and LC/MS) to monitor hydrolysis of cardiolipin by the four major types of PLA_2_, including GI and GV sPLA_2_, cPLA_2_, iPLA_2_ and Lp-PLA_2_ and also determined whether the hydrolysis occurs at the *sn*-1 and/or *sn*-2 positions. Additionally, we obtained further information regarding the interfacial catalytic activities of these PLA_2_ enzymes.

## Materials and Methods

### Materials

Natural and synthetic cardiolipin and 1-palmitoyl-2-arachidonoyl-*sn*-phosphatidylcholine (PAPC) were from Avanti Polar Lipids. 1-palmitoyl-2-arachidonyl- [arachidonyl-1-^14^C]–phosphatidylcholine was purchased from Perkin Elmer. All other reagents were analytical reagent grade or better.

### PLA_2_ Activity Assay

For the basal specific activity of PLA_2_, assays were performed in a buffer composed of 100 mM HEPES at pH 7.5. The mixed micelles were composed of 0.1 mM PAPC (containing ^14^C labeled PAPC with 80,000 cpm) and 0.4 mM Triton X-100 in a final volume of 500 µl. Different compositions of lipid mixed-micelles containing PAPC, cardiolipin and PI(4,5)P_2_ were mixed and dried, and then prepared utilizing the same method. ATP, Ca^2+^ and DTT were added depending on the requirement of the particular PLA_2_. GIA sPLA_2_ and GV sPLA_2_ assays were carried out in 5 mM Ca^2+^; GIVA cPLA_2_ assays were carried out in 3% PI(4,5)P_2_, 2 mM DTT and 0.1 mM Ca^2+^, and GVIA iPLA_2_ assays were carried out in 1 mM EDTA, 2 mM ATP and 4 mM DTT. The reaction was initiated by adding PLA_2_ to mixed micelles and incubated at 40°C for 30 min. After incubation, the reaction was quenched, and the fatty acids were extracted using a modified Dole assay protocol as previously described [38], [39].

### Fatty Acid Analysis by GC/MS

For GC/MS analysis, the deuterated internal standards were added before fatty acid extraction for the calibration of extraction efficiency and handling errors. The free fatty acids were analyzed as their pentafluorobenzyl (PFB) bromide derivatives in negative ionization mode [40]. The extracted free fatty acids were taken up in 25 µl of 1% diisopropylethylamine in acetonitrile and derivatized with 25 µl 1% PFB bromide in acetonitrile at room temperature for 20 min in capped glass tubes. The solvent was removed by vacuum evaporator, the residue was dissolved in 50 µl isooctane, and 1 µl of the PFB esters was analyzed by GC electron capture MS (GC/EC/MS). The fatty acid esters dissolved in 50 µl isooctane were injected (1 µl) into an Agilent 6890N gas chromatograph equipped with an Agilent 7683 auto-sampler (Santa Clara CA). The amounts of fatty acids were quantitated by the standard curve between 500 µg and 0.05 µg of fatty acids. Extraction and ionization efficiencies were determined for the internal standards by comparing the ion intensity of the samples with those of the extraction controls. The range of efficiency for a typical experiment was between 20 and 75%, with an average of around 50%. PLA_2_ activities were calculated by the amount of quantified fatty acids.

Cardiolipin, 1′,3′-Bis-[1,2-di-(9Z-octadecenoyl)-sn-glycero-3-phospho]-sn-glycerol, and its PLA_2_ hydrolysis products, monolyso-cardiolipin and dilyso-cardiolipin were purified by Bligh and Dyer lipid extraction methods [41]. The extracted cardiolipin, monolyso-cardiolipin and dilyso-cardiolipin were dried with nitrogen gas at room temperature. Samples were resuspended in 50% solvent A (water-acetonitrile- acetic acid; 70/30/0.02, v/v/v), and 50% solvent B (acetonitrile-isopropyl alcohol; 50/50, v/v). The cardiolipin sample was then injected into a tandem quadrupole mass spectrometer (ABI 4000 Q-Trap MS) by the direct infusion technique. The cardiolipin was analyzed via MS and precursor ion in negative ion mode. The electrospray voltage was −4.5 kV, and the turbo ion spray source temperature was 525°C. The precursor ions of oleic acid (m/z = 281.4) used nitrogen as a collision gas. The cardiolipin (m/z = 728.2), the monolyso-cardiolipin (m/z = 596.4) and the dilyso-cardiolipin (m/z = 463.9) were monitored in the negative mode during a precursor scan experiment.

## Results and Discussion

### PLA_2_ Activity Toward Cardiolipin

**Figure 1:**
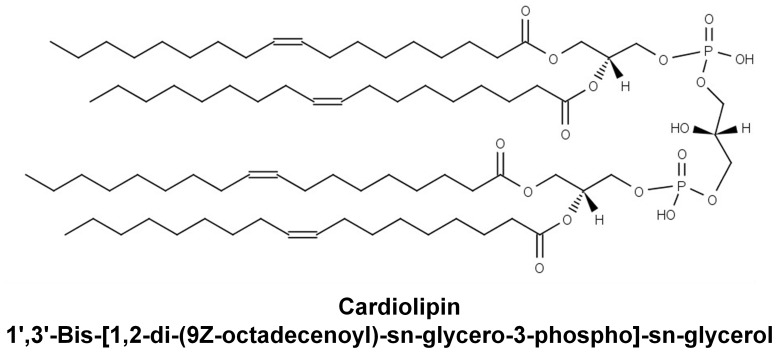
Structure of Cardiolipin. The structure of cardiolipin, 1′,3′-Bis-[1, 2-di-(9Z-octadecenoyl)-sn-glycero-3-phopho]-*sn*-glycerol, is drawn and adapted from LIPID MAPS (www.lipidmaps.org).

**Figure 2:**
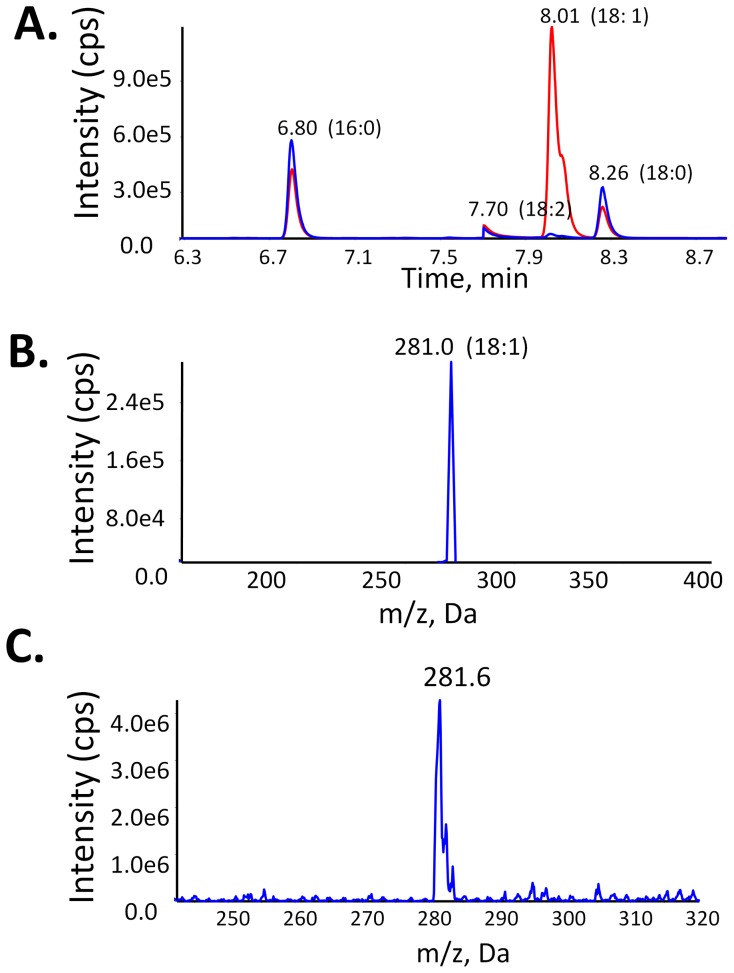
Cardiolipin Hydrolysis by iPLA_2_. A. GC/MS analysis of the free fatty acids released from cardiolipin by hydrolysis with (red) and in the absence of (blue) iPLA_2_. B. The main mass peak released by iPLA_2_ at 8.0 min on GC is oleic acid (18∶1). C. LC/MS analysis of the main fatty acid released from cardiolipin hydrolysis by iPLA_2_.

Cardiolipin has been shown to be a substrate for both cPLA_2_ and sPLA_2_ using fluorescence labeling substrate [7]. This indicates that the phospholipid *sn*-2 position can be recognized by phospholipase A_2_, despite its unique structure containing four fatty acyl chains with a bulky and negatively charged head group (Fig. 1). Whether cardiolipin is also a substrate for iPLA_2_ and Lp-PLA_2_ was not known. The direct measurement and comparisons of the PLA_2_ activity by following fatty acid hydrolysis has not been reported. Here we showed that the hydrolysis of tetraoleoyl(18∶1)-cardiolipin can be monitored by measuring the oleic acid released by both GC/MS and LC/ESI-MS/MS (Fig. 2) without fluorescent or radio-labeling. Three common contaminants from single-use glassware during fatty acid derivatization, 16∶0, 18∶2 and 18∶0 do not affect the quantification of 18∶1 on GC/MS. Because oleic acid (18∶1) is the most abundant fatty acyl chain in the biological samples, measurement of the oleic acid release is the best way to quantify cardiolipin hydrolysis. The advantage of the oleic acid measurement on LC-MS/MS is to avoid those fatty acid contaminants and the oleic acid peak can be isolated in the precursor mode.

**Figure 3:**
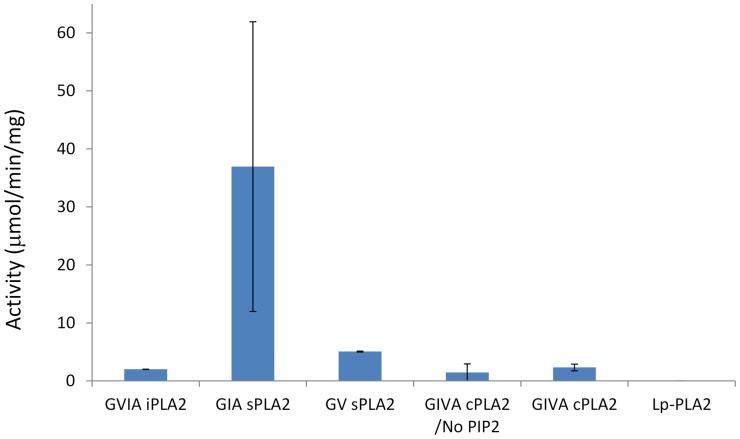
PLA_2_s Specific Activities toward Cardiolipin. The activities of GVIA iPLA_2_, GIA sPLA_2_, GV sPLA_2_, GIVA cPLA_2_ and Lp-PLA_2_, toward cardiolipin were examined by GC mass spectrometry. cPLA_2_ activities both with and without PIP_2_ activation are shown.

We further examined the cardiolipin activities of five different PLA_2_ enzymes representing four different PLA_2_ types by GC mass spectrometry (Fig. 3). The activities of these five highly purified enzymes, GVIA iPLA_2_, GIA sPLA_2_, GV sPLA_2_, GIVA cPLA_2_ and Lp-PLA_2_ have been well characterized previously [38], [39]. The results show that cardiolipin is a substrate for GVIA iPLA_2_, GIA sPLA_2_, GV sPLA_2_ and GIVA cPLA_2_, but not Lp-PLA_2_. The specific activities are 2.0 µmol/min/mg for GVIA iPLA_2_, 36.9 µmol/min/mg for GIA sPLA_2_, 5.0 µmol/min/mg for GV sPLA_2_ and 2.3 µmol/min/mg for GIVA cPLA_2_. The negatively charged PI(4,5)P_2_ binds to cPLA_2_ in a 1∶1 stoichiometry to increase its enzymatic activity [42]. A lysine pocket in the catalytic domain (Lys488, Lys541, Lys543, and Lys544) was shown to be essential for PIP_2_-dependent activity increases [43]. PIP_2_ therefore was recognized as an activator of cPLA_2_ and was included in the standard assay. Without PIP_2_ in the cardiolipin assay, GIVA cPLA_2_ showed an activity of 1.4 µmol/min/mg, consistent with the activation of cPLA_2_ by PIP_2_, though less so than with PAPC as substrate, suggesting perhaps that PIP_2_ may have less impact on the negatively charged surface of cardiolipin. Interestingly, GIA sPLA_2_ purified from snake venom shows the highest activity among all PLA_2_s. The sPLA_2_ in the snake venom can cause excessive inflammation when added to tissues through the hydrolysis of phospholipid membranes [44], [45].

### Lyso-cardiolipin Analysis by LC/MS

**Figure 4:**
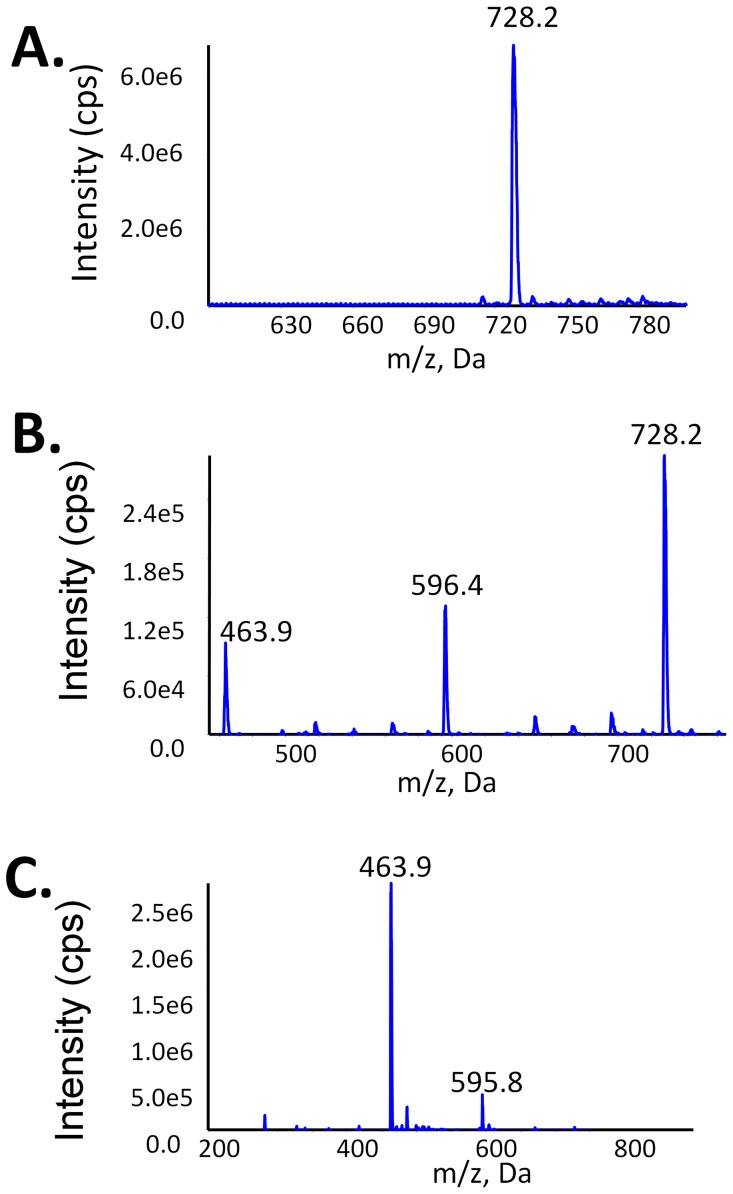
Cardiolipin and Lyso-cardiolipin Analysis by LC/MS. A. The dominant cardiolipin (18∶1) peak in mass spectrometry was observed as a doubly charged ion in the negative ion mode. B. Cardiolipin was hydrolyzed by iPLA_2_ in a mixed-micelle assay and the cardiolipin was extracted by the Bligh/Dyer method and then analyzed by LC/MS. C. GIA sPLA_2_ hydrolysis of cardiolipin results in the accumulation of mainly dilyso-cardiolipin.

The unique structure and the negative charge of cardiolipin stabilize the electron transport chain complex and maintain the structure of mitochondria membranes. Hydrolysis of cardiolipin will inevitably affect the structure and function of mitochondria. We have demonstrated that cardiolipin is a substrate for sPLA_2_, cPLA_2_ and iPLA_2_ (Fig. 2). The hydrolysis of cardiolipin should generate lyso-cardiolipin products and we employed tandem mass spectrometry to monitor the production of these species (Fig. 4).

The dominant fatty acyl on cardiolipin (18∶1) peak in mass spectrometry is seen as a doubly charged ion in the negative mode (Fig. 4A). These two negative charges of cardiolipin are from the two negative charges on the phosphate groups. The cardiolipin was hydrolyzed by iPLA_2_ in a mixed-micelle assay and the cardiolipin was extracted by the Bligh/Dyer method and then analyzed by LC/ESI-MS/MS (Fig. 4B). We identified the monolyso-cardiolipin (m/z = 596.4) and the dilyso-cardiolipin (m/z = 463.9) peaks by monitoring oleic acid (m/z = 281.4) in a precursor ion scan experiment. Both lyso-cardiolipins are also doubly charged as is cardiolipin. We have not detected any trilyso-cardiolipins, which would probably be very hydrophilic and not extracted by the current extraction method. Alternatively, iPLA_2_ may never cleave the *sn*-1 fatty acyl chain. Interestingly, sPLA_2_, which is known to have only *sn*-2 specific activity, only cleaves two fatty acyl chains off the cardiolipin resulting in the accumulation of dilyso-cardiolipin (Fig. 4C). This indicates the two *sn*-2 positions also have significantly higher rates of hydrolysis by PLA_2_ than the two *sn*-1 positions for sPLA_2_. We further purified the dilyso-cardiolipin from the GIA sPLA_2_ assay of cardiolipin. The hydrolyzed products have 90% dilyso-cardiolipin, which is the sn-1(1′)-diacyl-lysocardiolipin, and contain less than 10% of monolyso-cardiolipin; the latter may arise from migration of the sn-1 fatty acid to the sn-2 position as occurs with PC. Assaying the five phospholipase A_2_s for PLA_1_ activity against dilyso-cardiolipin gave no activity.

### Differential PLA_2_ Activities

The presence of four sterically distinct fatty acyl chains in cardiolipin presents challenges for kinetic studies of PLA_2_ hydrolysis. These phospholipase A_2_(s) have specific activity against the two *sn*-2 fatty acyl chains, but no activity toward the *sn*-1 chains. Previously, iPLA_2_ has been reported to catalyze a transacylase reaction [35]. Cardiolipin, monolyso-cardiolipin and dilyso-cardiolipin can be measured in one mass spectrometry run (Fig. 3). Here, we aimed at understanding how PLA_2_ hydrolyzes cardiolipin to produce lyso-cardiolipins over a 2-hour time scale.

**Figure 5:**
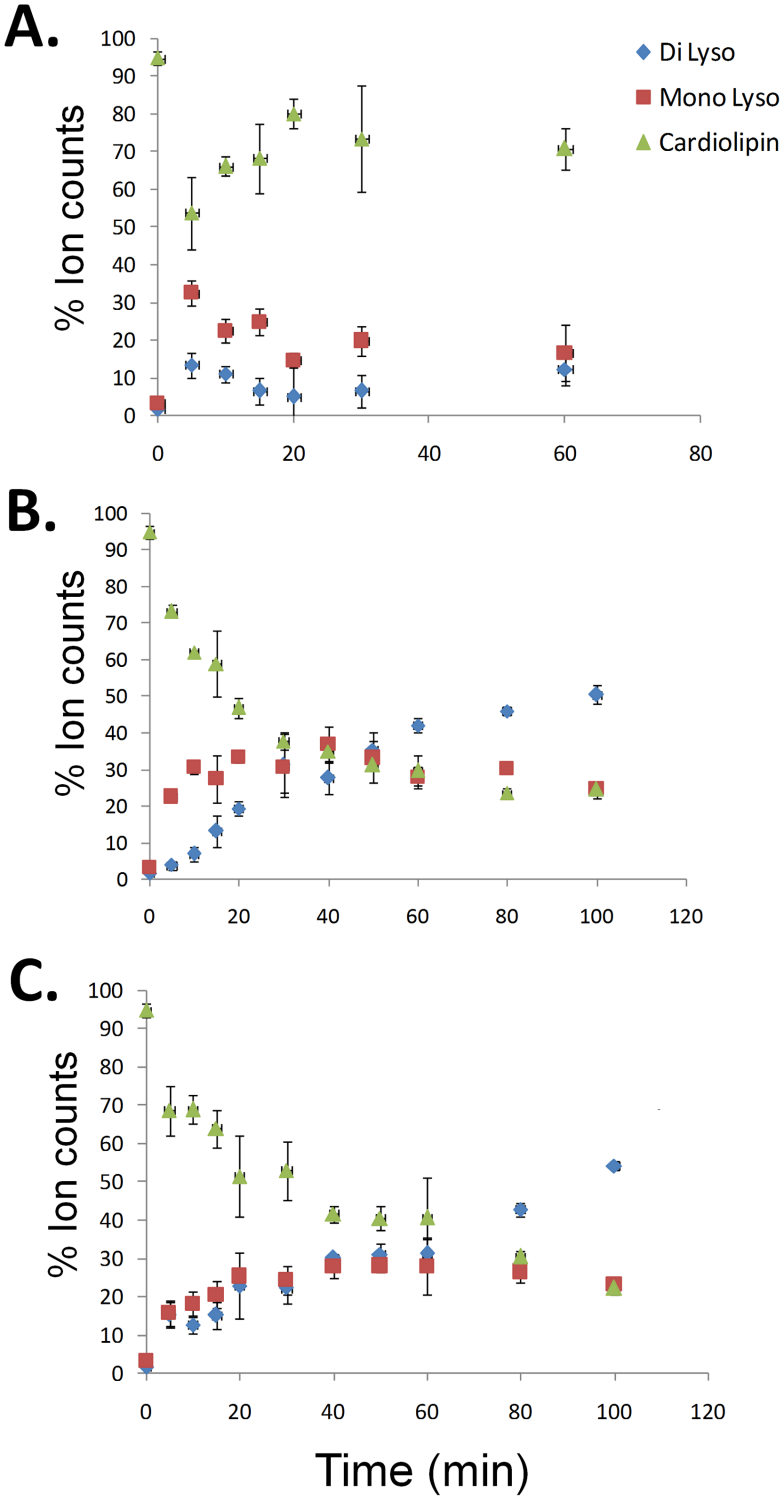
Differential Cardiolipin Hydrolysis by iPLA_2_ and sPLA_2_. The hydrolysis of cardiolipin by A. GVIA iPLA_2_, B. GIA sPLA_2_ and C. GV sPLA_2_ were examined in mixed micelle assays containing 100 µM cardiolipin and 400 µM Triton X-100 over a 100 min time course. The cardiolipin (green), monolyso- (red) and dilyso-cardiolipin (blue) were measured in the same samples by LC/MS. The percentages of cardiolipin and lyso-cardiolipin are based on ion intensity counts.

The mixed micelle assays contained 100 µM cardiolipin and 400 µM of Triton. The cardiolipin, monolyso-, and dilyso- cardiolipin were measured in the same samples by LC-MS/MS (Fig. 4B). The total ion counts for all cardiolipin species were calculated in each sample. The percentages of cardiolipin and lyso-cardiolipin were determined by the ratio of the ion counts for this particular cardiolipin species to the total ion counts (Fig. 5). The two sPLA_2_s showed a very similar pattern with the hydrolysis of cardiolipin occurring relatively early in the assay (Fig. 5B, C). The production of monolyso-cardiolipin increased during the initial 20 min and then reached a plateau. Dilyso-cardiolipin production continued through the whole time course. After 60 min, the dilyso-cardiolipin became the most abundant species. On the other hand, iPLA_2_ showed a completely different pattern. The cardiolipin percentage remained above 50% and lyso-cardiolipin levels stayed below 30%. The accumulation of dilyso-cardiolipin observed in the sPLA_2_ experiments did not occur for iPLA_2_ hydrolysis. This indicates that iPLA_2_ is regulated by a different mechanism than sPLA_2_ to maintain cardiolipin and lyso-cardiolipin at constant levels.

### Cardiolipin Effects on PLA_2_ Activities

**Figure 6:**
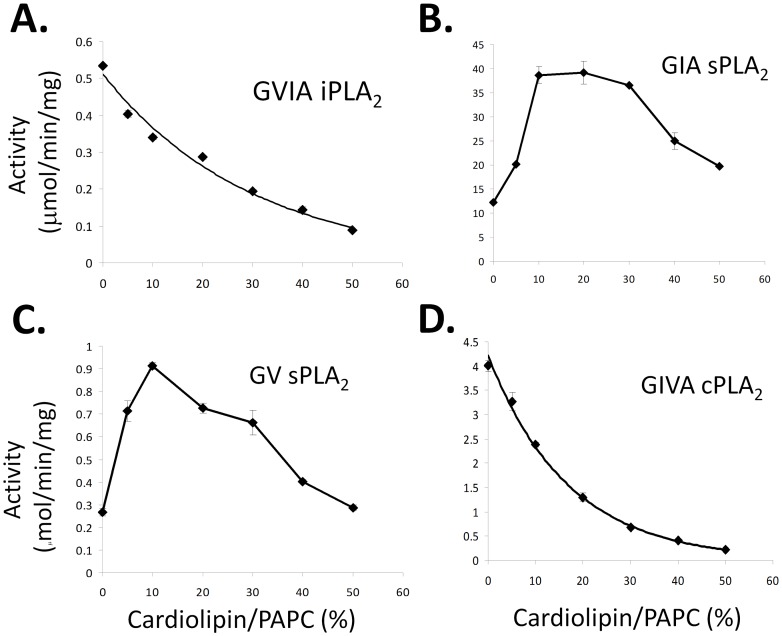
Cardiolipin Effects on PLA_2_ Activity toward PAPC. The *in vitro* mixed micelle assay was utilized to determine if cardiolipin affects the enzymatic activities of A. GVIA iPLA_2_, B. GIA sPLA_2_, C. GV sPLA_2_, and D. GIVA cPLA_2_ toward PAPC. Mixed micelles composed of 100 µM phospholipid and 400 µM Triton X-100 was employed as substrate containing the mole % of cardiolipin to PAPC indicated.

Cardiolipin is not only a substrate of various PLA_2_s, but it may also play a key role in regulating PLA_2_ activities at the membrane surface. The catalytic activities of PLA_2_ and phospholipid metabolism can be affected by the presence of the bulky and negatively charged cardiolipin. Hence, we have utilized an *in vitro* mixed micelle assay to determine the effect of cardiolipin on the enzymatic activities of five different PLA_2_s acting on PAPC (Fig. 6). 1-palmitoyl(16∶0)-2-arachidonoyl(20∶4)-*sn*-phosphatidylcholine (PAPC) was chosen because it does not interfere with the measurement of the oleic acid (18∶1) release from cardiolipin. A total of 100 µM phospholipid and 400 µM Triton X-100 was used as substrate. The results showed two major types of cardiolipin effects, activation or inhibition, on PLA_2_ activities. The inhibition by cardiolipin was observed for the activity of iPLA_2_ and cPLA_2_ toward PAPC (Fig. 6A, D). At 50% cardiolipin, iPLA_2_ activity was inhibited 80% and cPLA_2_ activity was inhibited 90%. The decrease caused by the addition of cardiolipin was not linear. The decreased level was higher than surface dilution of PAPC would predict assuming equal surface areas for Triton X-100, PC and cardiolipin molecules. Of course, the bulky volume of cardiolipin makes the calculation of surface dilution more complex than with simpler lipids in the PAPC/Triton X-100 mixed micelle system. In contrast, activation effects were observed with GIA and GV sPLA_2_ acting on PAPC which occurred when the phospholipid composition contained 0–20% cardiolipin (Fig. 6B, C). The PLA_2_ activity decreased when the cardiolipin content was above 20%. The decreased rate of activity was similar to that expected for surface dilution of the PAPC, but represents a complex mixture of effects. The maximum increase was 4 fold for both GIA sPLA_2_ and GV sPLA_2_. In the presence of 50% cardiolipin, the activity for both enzymes toward PAPC was at the same level as if cardiolipin was not added. Note that Lp-PLA_2_ did not show significant activity toward PAPC or cardiolipin confirming that it has specificity for short chain containing and/or oxidized phospholipids [46].

**Figure 7:**
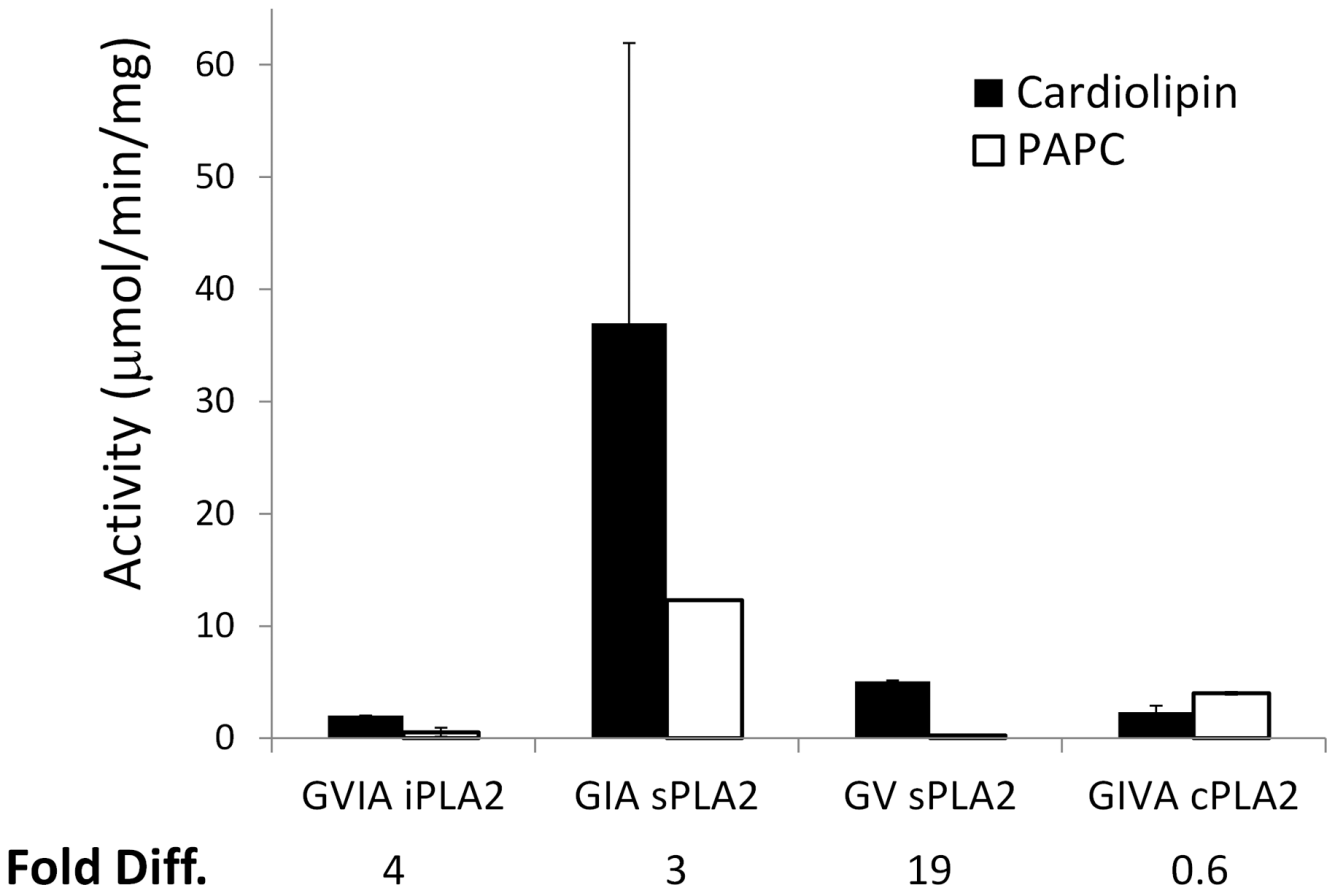
Comparison of PLA_2_ activities. The activities of GVIA iPLA_2_, GIA sPLA_2_, GV sPLA_2_, and GIVA cPLA_2_ toward cardiolipin (blue) and PAPC (red) are shown. The assays were conducted in mixed micelle assays containing either 100 µM cardiolipin or 100 µM PAPC and 400 µM Triton X-100. The fold differences between cardiolipin and PAPC were calculated and are shown.

Because both cardiolipin and PAPC are substrates for PLA_2_, the activation or inhibition curves could be altered by a surface charge effect, a competition effect and/or a surface dilution effect, we further examined the activity differences between these two substrates (Fig. 7). For iPLA_2_ and cPLA_2_, the activity decreased along with the increase of cardiolipin. The activity of iPLA_2_ toward cardiolipin was 2 µmol/min/mg, which was 3.8 times higher than toward PAPC in this assay. However, cPLA_2_ showed the opposite relative activity toward cardiolipin and PAPC. Under activating conditions, GIA sPLA_2_ showed a 3 fold greater activity toward PAPC than toward cardiolipin. GV sPLA_2_ showed a higher activity toward cardiolipin (18∶1) than toward the PAPC substrate. The competition and surface dilution effects did not appear significant when the overall cardiolipin was below 20%. When cardiolipin was increased above 20%, cardiolipin became a major substrate, and then the competition between cardiolipin and PAPC became apparent. sPLA_2_ is particularly interesting in that it has high activity against cardiolipin and cardiolipin presence can increase its activity toward phosphatidylcholine. Negative charge may play a key role of the increase of activity, suggesting the hydrolysis of the cardiolipin in mitochondria may speed up the breakdown of other phospholipids by any secreted PLA_2_ generated.

### Conclusions

Each of the five enzymes studied are thought to have a different physiological function and the PLA_2_ activities toward cardiolipin as a substrate are significantly different for each (Fig. 7). The unique structure of cardiolipin also differentially affects the PLA_2_ activities toward other phospholipids (Fig. 6). Two major features of cardiolipin are the large head group formed by two glycerol backbones and its two negative charges from the two phosphates. One would assume that the large head group would affect the uniform lipid surface and affect activity as seen with iPLA_2_ and cPLA_2_, but not sPLA_2_ and Lp-PLA_2_. Interestingly, cPLA_2_ and iPLA_2_ are the two enzymes with high molecular weights and both contain a regulatory domain and a catalytic domain. Even 5% cardiolipin can decrease their activity toward PC. The other complication is the surface dilution effect. The addition of cardiolipin dilutes the concentration of PAPC on the surface of the Triton X-100 mixed micelles. The inhibition toward cPLA_2_ is more significant than iPLA_2_. Note that the cPLA_2_ mixed micelle assay contains the negatively charged PIP_2_ and the surface dilution effects by cardiolipin may be enhanced by PIP_2_ dilution. On the other hand, sPLA_2_ is only affected above 20% cardiolipin. Interestingly, cardiolipin is predominantly found in mitochondria, which contains 10–20% cardiolipin. In the mixed micelle assays, the cardiolipin forms a negatively charged surface and sPLA_2_ has been shown to favor anionic phospholipid substrates. The increased affinity toward the anionic surface may play an important role. The surface dilution effect starts to take place and decrease the PLA_2_ activities when the cardiolipin content is above 20% in the GIA and GV sPLA_2_ assays. Overall, cardiolipin alters membrane dynamics, which affects these different enzymes binding to micelles and catalyzing lipid hydrolysis. The fatty acid composition at the *sn*-2 positions may also be a factor. Among these enzymes, sPLA_2_ and iPLA_2_ do not have much specificity for the specific fatty acid while cPLA_2_ is selective for arachidonic acid and Lp-PLA_2_ is very specific for short fatty acyl chains and oxidized fatty acids. Because the availability of commercial cardiolipins with different fatty acyl composition is limited at this time, we cannot yet address the specificity further.

## References

[1] Dennis EA, Cao J, Hsu YH, Magrioti V, Kokotos G (2011) Phospholipase A2 enzymes: physical structure, biological function, disease implication, chemical inhibition, and therapeutic intervention. Chem Rev 111: 6130–6185. 21910409 10.1021/cr200085w PMC3196595

[2] Buczynski MW, Dumlao DS, Dennis EA (2009) Thematic Review Series: Proteomics. An integrated omics analysis of eicosanoid biology. J Lipid Res 50: 1015–1038. 19244215 10.1194/jlr.R900004-JLR200 PMC2681385

[3] Six DA, Dennis EA (2000) The expanding superfamily of phospholipase A(2) enzymes: classification and characterization. Biochim Biophys Acta 1488: 1–19. 11080672 10.1016/s1388-1981(00)00105-0

[4] Pangborn MC (1947) The composition of cardiolipin. J Biol Chem 168: 351–361. 20291094

[5] Hirschberg CB, Kennedy EP (1972) Mechanism of the enzymatic synthesis of cardiolipin in Escherichia coli. Proc Natl Acad Sci U S A 69: 648–651. 4551982 10.1073/pnas.69.3.648 PMC426527

[6] Hostetler KY, van den Bosch H, van Deenen LL (1972) The mechanism of cardiolipin biosynthesis in liver mitochondria. Biochim Biophys Acta 260: 507–513. 4556770 10.1016/0005-2760(72)90065-3

[7] Buckland AG, Kinkaid AR, Wilton DC (1998) Cardiolipin hydrolysis by human phospholipases A2. The multiple enzymatic activities of human cytosolic phospholipase A2. Biochim Biophys Acta 1390: 65–72. 9487141 10.1016/s0005-2760(97)00170-7

[8] Marinetti GV (1964) Hydrolysis of Cardiolipin by Snake Venom Phospholipase A. Biochim Biophys Acta. 84: 55–59. 10.1016/0926-6542(64)90100-3 14124756

[9] Malhotra A, Edelman-Novemsky I, Xu Y, Plesken H, Ma J, et al (2009) Role of calcium-independent phospholipase A2 in the pathogenesis of Barth syndrome. Proc Natl Acad Sci U S A 106: 2337–2341. 19164547 10.1073/pnas.0811224106 PMC2650157

[10] Ascenzi P, Polticelli F, Marino M, Santucci R, Coletta M (2011) Cardiolipin drives cytochrome c proapoptotic and antiapoptotic actions. IUBMB Life 63: 160–165. 21445846 10.1002/iub.440

[11] Claypool SM (2009) Cardiolipin, a critical determinant of mitochondrial carrier protein assembly and function. Biochim Biophys Acta 1788: 2059–2068. 19422785 10.1016/j.bbamem.2009.04.020 PMC2757529

[12] Sparagna GC, Lesnefsky EJ (2009) Cardiolipin remodeling in the heart. J Cardiovasc Pharmacol 53: 290–301. 19276988 10.1097/FJC.0b013e31819b5461

[13] Gomez B Jr, Robinson NC (1999) Phospholipase digestion of bound cardiolipin reversibly inactivates bovine cytochrome bc1. Biochemistry 38: 9031–9038. 10413476 10.1021/bi990603r

[14] Eble KS, Coleman WB, Hantgan RR, Cunningham CC (1990) Tightly associated cardiolipin in the bovine heart mitochondrial ATP synthase as analyzed by 31P nuclear magnetic resonance spectroscopy. J Biol Chem 265: 19434–19440. 2147180

[15] Kalanxhi E, Wallace CJ (2007) Cytochrome c impaled: investigation of the extended lipid anchorage of a soluble protein to mitochondrial membrane models. Biochem J 407: 179–187. 17614790 10.1042/BJ20070459 PMC2049027

[16] Paradies G, Petrosillo G, Pistolese M, Di Venosa N, Federici A, et al (2004) Decrease in mitochondrial complex I activity in ischemic/reperfused rat heart: involvement of reactive oxygen species and cardiolipin. Circ Res 94: 53–59. 14656928 10.1161/01.RES.0000109416.56608.64

[17] Lesnefsky EJ, Slabe TJ, Stoll MS, Minkler PE, Hoppel CL (2001) Myocardial ischemia selectively depletes cardiolipin in rabbit heart subsarcolemmal mitochondria. Am J Physiol Heart Circ Physiol 280: H2770–2778. 11356635 10.1152/ajpheart.2001.280.6.H2770

[18] Paradies G, Petrosillo G, Ruggiero FM (1997) Cardiolipin-dependent decrease of cytochrome c oxidase activity in heart mitochondria from hypothyroid rats. Biochim Biophys Acta 1319: 5–8. 9107312 10.1016/s0005-2728(97)00012-1

[19] Ott M, Zhivotovsky B, Orrenius S (2007) Role of cardiolipin in cytochrome c release from mitochondria. Cell Death Differ 14: 1243–1247. 17431425 10.1038/sj.cdd.4402135

[20] Belikova NA, Vladimirov YA, Osipov AN, Kapralov AA, Tyurin VA, et al (2006) Peroxidase activity and structural transitions of cytochrome c bound to cardiolipin-containing membranes. Biochemistry 45: 4998–5009. 16605268 10.1021/bi0525573 PMC2527545

[21] Hughes GR (1983) Thrombosis, abortion, cerebral disease, and the lupus anticoagulant. Br Med J (Clin Res Ed) 287: 1088–1089. 10.1136/bmj.287.6399.1088 PMC1549319 6414579

[22] Harris EN, Gharavi AE, Asherson RA, Boey ML, Hughes GR (1984) Cerebral infarction in systemic lupus: association with anticardiolipin antibodies. Clin Exp Rheumatol 2: 47–51. 6442638

[23] Edwards T, Thomas RD, McHugh NJ (1993) Anticardiolipin antibodies in ischaemic heart disease. Lancet 342: 989. 10.1016/0140-6736(93)92035-r 8105234

[24] Rand JH, Wu XX, Andree HA, Ross JB, Rusinova E, et al (1998) Antiphospholipid antibodies accelerate plasma coagulation by inhibiting annexin-V binding to phospholipids: a “lupus procoagulant” phenomenon. Blood 92: 1652–1660. 9716593

[25] Deguchi H, Fernandez JA, Hackeng TM, Banka CL, Griffin JH (2000) Cardiolipin is a normal component of human plasma lipoproteins. Proc Natl Acad Sci U S A 97: 1743–1748. 10677528 10.1073/pnas.97.4.1743 PMC26506

[26] Ray NB, Durairaj L, Chen BB, McVerry BJ, Ryan AJ, et al (2010) Dynamic regulation of cardiolipin by the lipid pump Atp8b1 determines the severity of lung injury in experimental pneumonia. Nat Med 16: 1120–1127. 20852622 10.1038/nm.2213 PMC4500192

[27] Valianpour F, Mitsakos V, Schlemmer D, Towbin JA, Taylor JM, et al (2005) Monolysocardiolipins accumulate in Barth syndrome but do not lead to enhanced apoptosis. J Lipid Res 46: 1182–1195. 15805542 10.1194/jlr.M500056-JLR200

[28] Vreken P, Valianpour F, Nijtmans LG, Grivell LA, Plecko B, et al (2000) Defective remodeling of cardiolipin and phosphatidylglycerol in Barth syndrome. Biochem Biophys Res Commun 279: 378–382. 11118295 10.1006/bbrc.2000.3952

[29] Gadd ME, Broekemeier KM, Crouser ED, Kumar J, Graff G, et al (2006) Mitochondrial iPLA2 activity modulates the release of cytochrome c from mitochondria and influences the permeability transition. J Biol Chem 281: 6931–6939. 16407316 10.1074/jbc.M510845200

[30] Liou JY, Aleksic N, Chen SF, Han TJ, Shyue SK, et al (2005) Mitochondrial localization of cyclooxygenase-2 and calcium-independent phospholipase A2 in human cancer cells: implication in apoptosis resistance. Exp Cell Res 306: 75–84. 15878334 10.1016/j.yexcr.2005.01.011

[31] Seleznev K, Zhao C, Zhang XH, Song K, Ma ZA (2006) Calcium-independent phospholipase A2 localizes in and protects mitochondria during apoptotic induction by staurosporine. J Biol Chem 281: 22275–22288. 16728389 10.1074/jbc.M604330200 PMC1829309

[32] Song H, Bao S, Lei X, Jin C, Zhang S, et al (2010) Evidence for proteolytic processing and stimulated organelle redistribution of iPLA(2)beta. Biochim Biophys Acta 1801: 547–558. 20132906 10.1016/j.bbalip.2010.01.006 PMC2848069

[33] Ackermann EJ, Conde-Frieboes K, Dennis EA (1995) Inhibition of macrophage Ca(2+)-independent phospholipase A2 by bromoenol lactone and trifluoromethyl ketones. J Biol Chem 270: 445–450. 7814408 10.1074/jbc.270.1.445

[34] Lio YC, Reynolds LJ, Balsinde J, Dennis EA (1996) Irreversible inhibition of Ca(2+)-independent phospholipase A2 by methyl arachidonyl fluorophosphonate. Biochim Biophys Acta 1302: 55–60. 8695655 10.1016/0005-2760(96)00002-1

[35] Lio YC, Dennis EA (1998) Interfacial activation, lysophospholipase and transacylase activity of group VI Ca2+-independent phospholipase A2. Biochim Biophys Acta 1392: 320–332. 9630702 10.1016/s0005-2760(98)00049-6

[36] Hazen SL, Zupan LA, Weiss RH, Getman DP, Gross RW (1991) Suicide inhibition of canine myocardial cytosolic calcium-independent phospholipase A2. Mechanism-based discrimination between calcium-dependent and -independent phospholipases A2. J Biol Chem 266: 7227–7232. 2016324

[37] Zachman DK, Chicco AJ, McCune SA, Murphy RC, Moore RL, et al (2010) The role of calcium-independent phospholipase A2 in cardiolipin remodeling in the spontaneously hypertensive heart failure rat heart. J Lipid Res 51: 525–534. 19741254 10.1194/jlr.M000646 PMC2817582

[38] Barbayianni E, Stephens D, Grkovich A, Magrioti V, Hsu YH, et al (2009) 2-Oxoamide inhibitors of phospholipase A2 activity and cellular arachidonate release based on dipeptides and pseudodipeptides. Bioorg Med Chem 17: 4833–4843. 19443224 10.1016/j.bmc.2009.03.069 PMC2695835

[39] Kokotos G, Hsu YH, Burke JE, Baskakis C, Kokotos CG, et al (2010) Potent and selective fluoroketone inhibitors of group VIA calcium-independent phospholipase A2. J Med Chem 53: 3602–3610. 20369880 10.1021/jm901872v PMC2865582

[40] Pawlosky RJ, Sprecher HW, Salem N Jr (1992) High sensitivity negative ion GC-MS method for detection of desaturated and chain-elongated products of deuterated linoleic and linolenic acids. J Lipid Res 33: 1711–1717. 1464754

[41] Bligh EG, Dyer WJ (1959) A rapid method of total lipid extraction and purification. Can J Biochem Physiol 37: 911–917. 13671378 10.1139/o59-099

[42] Mosior M, Six DA, Dennis EA (1998) Group IV cytosolic phospholipase A2 binds with high affinity and specificity to phosphatidylinositol 4,5-bisphosphate resulting in dramatic increases in activity. J Biol Chem 273: 2184–2191. 9442060 10.1074/jbc.273.4.2184

[43] Six DA, Dennis EA (2003) Essential Ca(2+)-independent role of the group IVA cytosolic phospholipase A(2) C2 domain for interfacial activity. J Biol Chem 278: 23842–23850. 12672805 10.1074/jbc.M301386200

[44] Damerau B, Lege L, Oldigs HD, Vogt W (1975) Histamine release, formation of prostaglandin-like activity (SRS-C) and mast cell degranulation by the direct lytic factor (DLF) and phospholipase A of cobra venom. Naunyn Schmiedebergs Arch Pharmacol 287: 141–156. 49854 10.1007/BF00510446

[45] Cirino G, Peers SH, Wallace JL, Flower RJ (1989) A study of phospholipase A2-induced oedema in rat paw. Eur J Pharmacol 166: 505–510. 2806373 10.1016/0014-2999(89)90364-6

[46] Cao J, Hsu YH, Li S, Woods VL, Dennis EA (2011) Lipoprotein-associated phospholipase A(2) interacts with phospholipid vesicles via a surface-disposed hydrophobic alpha-helix. Biochemistry 50: 5314–5321. 21553808 10.1021/bi101916w PMC3110720

