# Correction: Assessing Phospholipase A_2_ Activity toward Cardiolipin by Mass Spectrometry

**DOI:** 10.1371/annotation/47607b18-ed69-4a08-8619-60c39bd83a13

**Published:** 2013-06-28

**Authors:** Yuan-Hao Hsu, Darren S. Dumlao, Jian Cao, Edward A. Dennis

The number of the second listed grant in Funding is incorrect. The correct grant number is: 20,501-38. 

